# A Multiscale Land Use Regression Approach for Estimating Intraurban Spatial Variability of PM_2.5_ Concentration by Integrating Multisource Datasets

**DOI:** 10.3390/ijerph19010321

**Published:** 2021-12-29

**Authors:** Yuan Shi, Alexis Kai-Hon Lau, Edward Ng, Hung-Chak Ho, Muhammad Bilal

**Affiliations:** 1 Institute of Future Cities (IOFC), The Chinese University of Hong Kong, Hong Kong, China; 2 Division of Environment and Sustainability, The Hong Kong University of Science and Technology, Clear Water Bay, Kowloon, Hong Kong, China; alau@ust.hk; 3 Department of Civil and Environmental Engineering, The Hong Kong University of Science and Technology, Clear Water Bay, Kowloon, Hong Kong, China; 4 Institute for the Environment, The Hong Kong University of Science & Technology, Clear Water Bay, Kowloon, Hong Kong, China; 5 School of Architecture, The Chinese University of Hong Kong, Hong Kong, China; edwardng@cuhk.edu.hk; 6 Institute of Environment, Energy and Sustainability (IEES), The Chinese University of Hong Kong, Hong Kong, China; 7 Department of Urban Planning and Design, The University of Hong Kong, Hong Kong, China; hcho21@hku.hk; 8 Lab of Environmental Remote Sensing (LERS), School of Marine Sciences, Nanjing University of Information Science and Technology, Nanjing 210044, China; muhammad.bilal@connect.polyu.hk

**Keywords:** PM_2.5_, spatial variability, geographic information system, multiscale, multi-source datasets

## Abstract

Poor air quality has been a major urban environmental issue in large high-density cities all over the world, and particularly in Asia, where the multiscale complex of pollution dispersal creates a high-level spatial variability of exposure level. Investigating such multiscale complexity and fine-scale spatial variability is challenging. In this study, we aim to tackle the challenge by focusing on PM_2.5_ (particulate matter with an aerodynamic diameter less than 2.5 µm,) which is one of the most concerning air pollutants. We use the widely adopted land use regression (LUR) modeling technique as the fundamental method to integrate air quality data, satellite data, meteorological data, and spatial data from multiple sources. Unlike most LUR and Aerosol Optical Depth (AOD)-PM_2.5_ studies, the modeling process was conducted independently at city and neighborhood scales. Correspondingly, predictor variables at the two scales were treated separately. At the city scale, the model developed in the present study obtains better prediction performance in the AOD-PM_2.5_ relationship when compared with previous studies ($\bar{R^{2}}$ from 0.72 to 0.80). At the neighborhood scale, point-based building morphological indices and road network centrality metrics were found to be fit-for-purpose indicators of PM_2.5_ spatial estimation. The resultant PM_2.5_ map was produced by combining the models from the two scales, which offers a geospatial estimation of small-scale intraurban variability.

## 1. Introduction

Poor air quality has been a major urban environmental issue in cities, especially those large and compact cities in Asia, for the last several decades [1]. Urbanization alters the local climate, thus contributing to ambient air pollution levels in cities [2]. The interaction between the urban environment and air pollution dispersion is a complex multiscale mechanism [3]. The spatial scales of atmospheric pollution can range from a few hundred meters for urban street canyons to a few hundred kilometers for a whole Megalopolis [4]. At the mesoscale, the spatial layout of artificial urban land cover types changes the near-surface aerodynamic roughness, which interacts with the atmospheric circulation, and consequently, alters the transportation of air pollutants [5]. At the city scale, urban land use planning changes the spatial distribution of the emission sources of air pollutants. As a result, the air quality situation in large cities is not homogenously distributed. A spatially heterogeneous urban environment (different combinations of building density and functions, road network, open space, etc.) makes the concentration levels of air pollutants vary from place to place [6]. At the neighborhood scale, the geometrical shapes of building clusters, street canyons, the arrangement of street trees, and vegetation are all influential factors of street-level air quality [7,8]. The above multiscale phenomenon creates a high-level spatial variability of pollution exposure levels in the urban environment. Therefore, investigating the multiscale complexity and fine-scale spatial variability of air pollution is challenging [9].

There are two types of air pollution modeling methods—deterministic and statistical [10]. Deterministic methods (numerical and computational modeling) are most commonly used in the modeling of mesoscale or city-scale air pollution. In the last few decades, various numerical and computational models have been developed from the perspective of atmospheric chemistry and physics [11,12]. Most of the Lagrangian and Eulerian grid dispersion models provide hourly-resolved fine temporal resolution but at a coarse spatial resolution of several kilometers [13,14]. Computational fluid dynamics (CFD) enables the accurate simulation of the movement of air pollutants at a very fine spatiotemporal scale. CFD is mostly used for air pollution investigation in street canyons [15] or city blocks [16] as it can only cover a limited spatial extent due to the high demands for computational powers and time costs. However, for air quality management and health risk assessment nowadays, the air pollution data needs to have both a relatively large spatial extent (i.e., an entire city) and a sufficiently high spatial resolution (sometimes, with a grid cell size of fewer than one hundred meters) to adequately capture small-scale intraurban spatial variability [17]. With geographical information systems (GIS), statistical methods offer data with a finer spatial resolution to help cater to the above need.

Statistical methods employ and combine in-situ monitored data from various sources, which can provide concise and quantitative information on spatiotemporal variability. Most of the routine air quality monitoring networks have long-term air quality data at an hourly-resolved temporal scale. Mobile monitoring techniques based on portable sensors capture fine-scale spatial variability [18,19]. AOD-ground-level PM_2.5_ correlation analysis is also a popular method [20,21] as the Satellite-derived aerosol observations provide extensive spatial coverage and temporal continuity [22]. Land-use regression (LUR) models from exposure science research provide fit-for-purpose predictions on long-term concentration at a fine spatial scale [23,24,25,26]. By combining the above data sources and techniques, studies on the development of exposure models have been conducted and show promising results [27,28]. Moreover, developing multiscale exposure models is also found to be a feasible way of capturing the intraurban spatial variability in air pollution distribution [29]. Such models can estimate air pollution concentration levels for large-scale spatial coverage while capturing small-scale spatial variability, which not only provides useful information on spatial exposure but also enables a quantitative understanding of the multiscale influence of urbanization on air pollution dispersion in cities. In addition, it also helps with the identification of influential factors of intraurban spatial variability of air pollution. In this study, aiming at tackling this multiscale complexity in the spatial variability of air pollution affecting pedestrians’ exposure levels, we propose a multiscale LUR model and offer a geospatial estimation of small-scale intraurban variability of air pollution. Particularly, we focus on exposure to PM_2.5_ (particulate matter with an aerodynamic diameter of less than 2.5 µm). A geospatial approach—multiscale LUR modeling that combines various types of multiscale data sources was adopted to estimate the intraurban spatial variability of PM_2.5_ exposure in the compact built environment using the city of Hong Kong as a study area. Unlike most LUR and AOD-PM_2.5_ studies, the modeling process was conducted independently at city and neighborhood scales. Correspondingly, predictor variables at the two scales were treated separately. Moreover, point-based building morphological indices and road network centrality metrics were also examined in the modeling process, which is rare in other existing LUR studies.

## 2. Materials and Methods

### 2.1. Study Area

**Figure 1:**
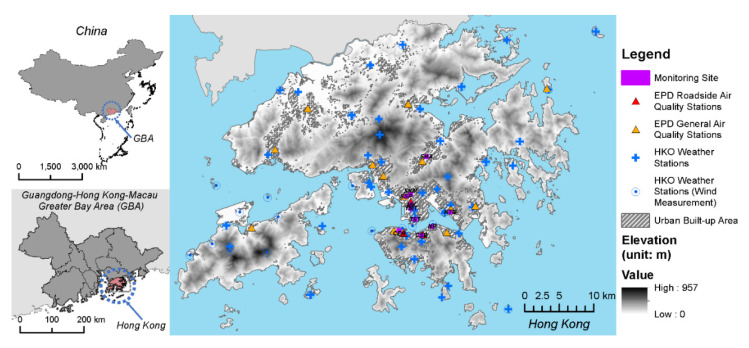
The air quality monitoring network, weather stations for wind measurement, and site selection in this study.

Hong Kong (Figure 1), a highly urbanized large city with serious air pollution, was selected as the testbed city for developing multiscale LUR models of PM_2.5_ exposure. Hong Kong is one of the world’s most compact cities with a large population of over seven million people living in a total land area of 1100 km^2^. Its population density is around 6700 persons/km^2^. Like most other large cities in the world, Hong Kong also has air pollution issues. The highly complex and diverse urban environment makes Hong Kong an ideal study area for the development of multiscale LUR models.

### 2.2. PM_2.5_ and AOD Data

#### 2.2.1. Satellite-Derived AOD Data at City Scale

In this study, MODIS Multi-Angle Implementation of Atmospheric Correction (MAIAC) AOD (MCD19A2) was used to represent the spatial distribution of surface PM_2.5_ concentration over the study area corresponding to the period of ground monitoring and sampling (described in Section 2.2.2 and Section 2.2.3). Compared with the 3-km MODIS product [30], MAIAC has a satisfying accuracy at a finer spatial resolution (1-km) and higher spatial coverage of retrieval [31]. Therefore, MAIAC AOD is more suitable for air quality studies, notably, it is better at providing details of fine-scale aerosol characteristics over study areas with heterogeneous geographic context. A QA filter should be applied to select the best-quality MAIAC AOD data [31]. Most applications use the filter of QA.CloudMask = “Clear” in order to ensure a high quality of the retrieved AOD. However, it has been identified that in some locations with high spatial aerosol variability, this filter may systematically erase AOD retrievals in cloud-free conditions over certain urban areas [32]. In such cases, the following filters to define high-quality AOD retrievals: QA.CloudMask = “Clear” or “Possibly cloudy”. Furthermore, pixels with the filter of QA for AOD = “AOD within ±2 km from the coastline” were also included to extend the spatial coverage of prediction, as there are major urban built-up areas in Hong Kong within 2 km from the coastline (can be seen in Figure 1). MAIAC jointly processes MODIS Terra and Aqua sensors as a single sensor due to the Terra-to-Aqua cross-calibration of the entire C6 MODIS data collection [33]. In the data collection, most daily files contain multiple orbit overpasses for combined Terra and Aqua. Data from Aqua and Terra were combined and averaged to provide an estimation of the daily average AOD value [34]. Chemical transport models, AOD validation, and inter-comparison commonly use the input which is standardized to 0.55 μm [35]. Therefore, to keep the consistency, AOD retrieved at 0.55 µm was selected by this study.

#### 2.2.2. Long-term PM_2.5_ Monitoring Data at City Scale

During the study period (years 2018 and 2019), data from a total of 16 stations were available in the local air quality monitoring network in the study area (Figure 1). The stations in the network are located at representative places that cover various combinations of land use, road traffic network, natural topography (information shown in Table S1 of the Supplementary Materials). These stations are operated and regularly maintained by the local authority—Hong Kong Environmental Protection Department (HKEPD). The data archive of the hourly concentration level of PM_2.5_ is openly available to the public. The monitoring data from the network are readily comparable to air quality data from other cities, as the network has been recognized by the United Nations Environmental Programme (UNEP) and complies with international standards [36]. Therefore, we selected this data source for investigating the city-scale spatial variability of PM_2.5_.

#### 2.2.3. In-Situ PM_2.5_ Sampling at Neighborhood Scale

Sampling site selection: The city of this study is a large city with a compact and complex urban environment. The spatial variability in air pollution affecting pedestrians’ exposure levels in such a spatially heterogeneous city context cannot be fully represented by the sparsely located 16 air quality stations operated in the local air quality monitoring network. Therefore, this study selected ten study sites for air pollution sampling based on not only land use and natural topography but also district block function, building density and morphology, and road network layout. The ten sampling sites selected are representative of the diverse urban contexts of Hong Kong. The details of the ten selected sites are shown in Table S2 of the Supplementary Materials.

Sampling routes: The present study adopted a PM_2.5_ sampling method which was tested in a previous pilot study on small-scale spatial variability of pedestrian level particulate matter in three downtown commercial districts [37]. To investigate the spatial variability of PM_2.5_ exposure levels, one person kept strolling and rambling in a selected site area at a common pedestrian walking speed of 3 km/h (0.8 m/s) [38] with a backpack sampling unit (see the next paragraph—Sampling instrumentation). The sampling routes are shown in Figure S1 of the Supplementary Materials.

Sampling instrumentation: The instrument used in the previous study is an all-in-one backpack sampling unit assembled by combining various monitors and sensors. In the present study, the instrument was further improved, becoming smaller in size and more lightweight. The concentration levels of PM_2.5_ were continuously monitored using the TSI SidePak^TM^ personal aerosol monitor AM520 (a more portable laser-scattering device that is dedicatedly designed for assessing personal exposure to PM) instead of the DUSTTRAK^TM^ model 8534 monitor. The AM520 was set to sample and log PM_2.5_ concentration data with the time interval of 1 s (with this sampling interval, the spatial interval of sampling data points was approximately 1 m which is a fine resolution for spatial mapping and exposure assessment). A sample extension tube was connected to the inlet of AM520 to extend the inlet to the height of 1.6 m above the ground. During the measurement, the person who carried the backpack sampling unit stayed away from any random pollution sources nearby (e.g., smoking people, roadside vendors) in order to minimize random noise introduced in the data. Ambient air temperature (*T_a_*, °C) and relative humidity (*RH*, %) were synchronously sampled by a set of Testo 480 data logger and humidity/temperature probe. The *RH* data were used for the sampled PM_2.5_ data calibration. The AM520 sampled data were corrected using synchronously sampled *RH* based on the following equation [39]:

(1) $$Correction\;Factor=1+0.25\frac{RH^{2}}{\left(1-RH\right)}$$

The Global Positioning System (GPS) receiver was used to label the geolocation of each sample in the WGS 1984 coordinate system. The data logging time stamps were synchronized to Coordinated Universal Time (UTC).

*Sampling periods:* During the warm season (May to September) of Hong Kong, local emission sources dominate the local condition of PM_2.5_ [40], which minimizes the overwhelming effects of high background concentration levels caused by the long-distance transport of non-local emission sources. Therefore, in-situ measurements were conducted during the warm season (May to September) of the year 2018 and 2019 (the measurement date are shown in Table S2 of the Supplementary Materials). This experimental design simulated a scenario of typical city pedestrians’ outdoor activities. The sampling campaign was conducted in three different time slots (9 a.m.–11 a.m., 2 p.m.–4 p.m., and 7 p.m.–9 p.m.) each day to cover different periods and situations of traffic and human activities in a day. To investigate the spatial variability, day-to-day and hour-to-hour temporal variation in the measurement, data were adjusted based on the background air quality monitoring station of Hong Kong using the adjustment method that has been tested and adopted in a previous study of vehicle-based mobile measurement [41].

### 2.3. Spatial and Temporal Predictor Data

In this study, multiple data sources were collated to a predictor variables dataset for the multiscale LUR modeling process. Table 1 summarizes the predictor variables and the corresponding data sources used in the multiscale LUR modeling of this study.

#### 2.3.1. Weather Data and Sounding Data at City Scale

Incorporating meteorological information for exposure analysis helps with explaining the small-scale spatial variability in air pollution [43]. Particularly, incorporating interpolated observed wind information from a weather station network improves the model prediction performance [44,45]. In this study, near-surface meteorological data (i.e., air temperature, relative humidity, wind speed, rainfall, mean sea level pressure) were retrieved from the Hong Kong Observatory (HKO) weather monitoring network (Figure 1). Kriging, as one of the mostly-used geostatistical methods for meteorological applications [46], was performed to generate spatially interpolated meteorological data layers that cover the entire study area using a fine spatial resolution of 10 m, which is consistent with the land use information of Hong Kong introduced in the next section.

#### 2.3.2. Land-Use, Population, Road Network at City Scale

At the city scale, the distribution of residential zones, population density, the location of industrial and commercial point sources, traffic-related line sources on road networks are all factors that influence the PM_2.5_ spatial variability. The circular buffering method was used for quantifying the above influential factors and creating predictor variables (e.g., the total area of industrial zones, road network density, or population density within the circular buffer, etc.). The predictor variables were calculated in a series of circular buffers around the location of each long-term monitoring station. The land-use zoning information is publicly available from the local city planning authority—Planning Department (in the form of a land use map at 10 m resolution). The original complicated land-use zoning was reclassified to a five-class scheme: residential zone, commercial zone, industrial zone, governmental and facilities, greening, and open space, which makes the present study consistent and comparable to existing studies [24]. Spatial data of urban road networks, location of car parks, bus stops, and other transport facilities were extracted from Open Street Map (OSM). The population density map at 100 m resolution was derived from the WorldPop dataset [47].

#### 2.3.3. Road Segment Centrality and Accessibility as Proxy of Traffic Distribution

Traffic distribution is usually measured as the volume of vehicles passing through a road segment. A higher traffic volume leads to more traffic-related pollutant emissions. However, obtaining a full record of traffic volume that covers all road segments of a large network usually needs intensive resources or technical input. Regression modeling has been enlisted as a more economically feasible way of estimating traffic distribution [48,49], in which topological measures are an essential set of predictors. The network centrality, as a key factor of network analysis, has been used to investigate the characteristics of the urban roadways [50] and employed in the estimation of traffic distribution [51,52]. The Space Syntax approach is another road network topology analysis method based on the accessibility of road segments that has been used in understanding the traffic as well [53,54,55]. Based on the literature, in this study, we selected and calculated three centrality metrics and five spatial syntax metrics (as shown in Table 1) for all road segments in the study area using the Urban Network Analysis Tool [56] and Axwoman [57], respectively. These metrics were used as predictor variables in the neighborhood-scale modeling.

#### 2.3.4. Building Morphological Data at Neighborhood Scale

In the highly urbanized context, urban ventilation depends largely on the geometrical characteristics of buildings, which have a greater influence on the spatial variability of urban pollution [58]. A previous local study found that the pollutant concentration in street canyons had a strong and significant correlation with specific building morphological parameters [41]. In this study, building morphological parameters at both city-scale and neighborhood-scale were adopted for building multiscale models. At the city scale, the frontal area index (*FAI*), as a commonly-used wind direction-dependent measure of evaluating urban ventilation and a widely recognized influencing factor of air quality [59], was calculated using the following equation:

(2) $$FAI=\overset{8}{\underset{\theta =1}{\sum }}\left[\frac{A_{F\left(\theta \right)}}{A_{T}}\right]\cdot P_{\left(\theta \right)}$$

where, $A_{T}$ is the lot area of the land parcel. $A_{F\left(\theta \right)}$ is the total projected area along a particular wind direction (*θ*) of all buildings in the land parcel. *P*(*θ*) is the wind direction probability of the eight corresponding principal wind directions (*θ*). Similar to other city-scale predictor variables, the averaged *FAI* value within circular buffers around each long-term monitoring station was calculated. At the neighborhood scale, two point-based building morphological parameters, which are sky view factor (*SVF*) [60] and point-based *FAI* [61] were calculated for all recorded locations of the in-situ PM_2.5_ sampling data. *SVF* has been found associated with air quality [62] and calculated in the present study by using the method proposed by Dozier and Frew [63] which is shown in the equation below:

(3) $$SVF=\frac{1}{2\pi }\overset{2\pi }{\underset{0}{\int }}\left[\cos\beta \cos^{2}\varphi +\sin\beta \cdot \cos\left(\Phi -\alpha \right)\cdot (90-\varphi -\sin\varphi \cos\varphi )\right]d\Phi$$

where *SVF* is calculated for each location of the digital surface model (DSM, a raster layer contains elevation value of buildings and ground surface at each pixel) of sampling sites with slope aspect *α* and angle *β* based on the horizon angles *φ* in azimuth directions Φ of the hemisphere circle with the radius *d*. Point-based *FAI* ($FAI_{Point}$) is a fine-scale building morphological parameter developed for the fit-for-purpose estimation of the pedestrian-level wind speed at a high spatial resolution within urban areas [61]. Compared with conventional computational fluid dynamics (CFD) simulation, $FAI_{Point}$ is much more cost-effective as it can estimate pedestrian-level wind speed for a large spatial extent (i.e., the entire city) in almost real-time. Wind speed has been commonly regarded as a proxy of pollutants concentration level in the investigation of spatial variability in exposure [64] as stagnation of air in compact building clusters is often associated with elevated concentration of air pollutants and increased exposure to air pollution [65,66]. $FAI_{Point}$ is an extension of *FAI*. Therefore, their calculation shares common ground. It is calculated using the equation below:

(4) $$FAI_{Point}=\iint w_{l}[\overset{8}{\underset{\theta =1}{\sum }}\frac{A_{F\left(\theta ,x,y\right)}}{A_{T,circle}}\cdot P_{\left(\theta \right)}]dl/A_{T,circle}\;w_{l}=\left[0,\;1\right]$$

(5) $$w_{l}={\left(\frac{R-l}{R}\right)}^{c}\;c=2,\;l=\left[0,\;200\right]$$

where $A_{T,circle}$ is the circular area using a radius of R=200 m and the location of the test point (x,y) as the center of the circle. $A_{F\left(\theta ,x,y\right)}$ is calculated for each location in the circular area. An exponential (c=2) decay weighting factor (Equation (5)) is applied to represent the decreasing effect of the roughness elements along with the increase in the distance l between roughness elements and the test point [67].

### 2.4. Multiscale Land Use Regression Modeling

As mentioned in the introduction of the study, we aimed to develop a multiscale LUR model which enables a cost-efficient geospatial estimation of small-scale intraurban variability of intraurban air pollution for the study area. Therefore, the LUR modeling was conducted at two different scales. First, we performed city-scale LUR modeling based on long-term monitoring and city-scale predictors. This first stage LUR model provides an overview of the seasonal average concentration level of PM_2.5_ over the spatial extent. Then, we performed the second-stage LUR at a finer spatial scale using the in-situ PM_2.5_ sampling data neighborhood-scale predictors. The second-stage LUR model explains the fine-scale spatial variability which was used to downscale the city-scale spatial PM_2.5_ map from the first stage LUR modeling.

#### 2.4.1. City-Scale LUR Modeling and Mapping

The first stage LUR modeling provided an overview of the spatial distribution of the four seasonal average concentration levels of PM_2.5_ for the city. The development of city-scale LUR models consisted of three steps, which were (i) variable selection, (ii) regression modeling, and (iii) spatial mapping. At this step, instead of conventional multiple linear regression (MLR), we adopted geographically and temporally weighted regression (GTWR) modeling by using the predictor variable subset derived above. GTWR considers spatial and temporal variability, as it has been found that geographically weighted models perform better than MLR in the investigation of the effects of land use on urban air pollution variations [68,69,70]. The general model structure can be represented as follows:

(6) $$PM_{2.5ij}\;\sim \;\left(\alpha_{0}+\beta_{ij}\right)+\left(\alpha_{1}+\beta_{ij}\right)\times AOD_{ij}+\alpha_{2ij}P_{0ij}+\alpha_{3ij}P_{1ij}+\cdots \;+\alpha_{k+2ij}P_{kij}+\varepsilon$$

where *AOD_ij_* is the observed AOD at location *i* on time *j*, which is forced to be included in the model. $\left(\alpha_{0}+\beta_{ij}\right)$ is the intercept of the model.$\;\left(\alpha_{1}+\beta_{ij}\right)$ is the slope of *AOD_ij_*. $\alpha_{2ij}$,$\;\alpha_{3ij}$,…,$\;\alpha_{k+2ij}$ are the slopes for city-scale predictors *P_0ij_*, *P_1ij_*,…, *P_kij_* (that are derived from weather data, land use, population, and road network density) at location *i* on time *j*, which are all geographically and temporally varied. ε is residuals. First, all city-scale spatial predictor variables described in Table 1 were examined to screen out a subset of variables that could result in the best-performing model. Generally, the selection of buffer radius is based upon the decay correlation with the modeled pollutant [24]. The distance-decay curve method [71] that has always been widely used so far [72,73] was adopted by the present study to identify appropriate spatial predictor variables and the corresponding associated buffer distance. Specifically, the buffer radius with the highest correlation coefficient between the PM_2.5_ level and spatial predictor variables was identified, and the variables at their corresponding buffer were selected for further stepwise regression analysis. Then, the spatial predictor variables at their optimal buffer and all temporal variables were put together as the input for a stepwise regression analysis to derive a subset of variables that could result in the best-performing model. In this study, the stepwise regression yielded the following rules: (1) *p*-value < 0.05 for all variables included in the model; (2) variance inflation factor (VIF) < 3 for all variables to minimize the collinearity and overfitting issues. The predictor subset derived from the above stepwise regression was used to build seasonal GTWR models. Finally, based on the spatiotemporal GTWR models, we generated the seasonal averaged PM_2.5_ spatial map. AOD data (with an original resolution of 1 km) was smoothed with the bilinear interpolation to keep the resolution consistent with the land use data layers and interpolated meteorological data layers. The modeling and mapping results are shown in Section 3.1.

#### 2.4.2. Neighborhood-Scale LUR Modeling

To incorporate the neighborhood-scale spatial variability of PM_2.5_ concentration, we further developed the second stage LUR model using the data from in-situ sampling at the ten sites (refers to Section 2.2.3). All neighborhood-scale spatial variables (mentioned in Table 1) were used as predictors. The same variable selection method and criteria used in the first stage modeling were also adopted here. Instead of predicting the absolute value of in-situ sampling PM_2.5_ data, the neighborhood-scale LUR modeling focused on investigating the variation among different points in a site. Therefore, the variation in PM_2.5_ ($\Delta PM_{2.5}$) was used as the response variable. $\Delta PM_{2.5}$ is the difference between the measured PM_2.5_ concentration at a point and the site averaged PM_2.5_ (which is the mean of all measurement points in the entire site). The $\Delta PM_{2.5}$ value will be positive if the measured concentration at a point is higher than the site average, vice versa. The model structure is as follows:

(7) $$\Delta PM_{2.5}\;\sim \;\beta +\alpha_{1}P_{1}+\alpha_{2}P_{2}+\alpha_{3}P_{3}+\cdots +\alpha_{n}P_{n}+\varepsilon$$

where $\alpha_{1}$,$\;\alpha_{2}$,…,$\;\alpha_{n}$ are the slopes for city-scale predictors *P_1_*, *P_2_*,…, *P_n_*. As to the regression modeling, we used MLR instead of GTWR for the development of the neighborhood-scale model, as the in-situ PM_2.5_ sampling data mainly reflects the microscale effect. The microscale effect that happens in street canyons depends on the environmental condition of the specific road segment, which is supposed to be geolocation-independent. The $\Delta PM_{2.5}$ was calculated for all road segments. A road segment was treated as a line segment for the calculation of centrality and accessibility (described in Section 2.3.3), and the calculated values were spatially assigned to all locations within the street canyon corresponding to the road segment in the process of spatial mapping. As the model estimates the variation in PM_2.5_ instead of the absolute concentration value, the resultant map had a data distribution across positive and negative. It is important to note that the regression model is only valid within the spatial range of road and street area (the centrality and accessibility variables representing traffic were only calculated at the road network) and the numerical range of input predictor variables (no “extrapolation” [74] was done for the model to reduce the uncertainty). The resultant map reflects the variation at a finer spatial scale, but it does not have full spatial coverage of the city. The valid part of PM_2.5_ variability estimation was overlayed together with the city-scale PM_2.5_ map from Section 2.4.1 in order to provide the final PM_2.5_ concentration map.

## 3. Results

### 3.1. City-Scale LUR Modeling and Mapping Results

**Figure 2:**
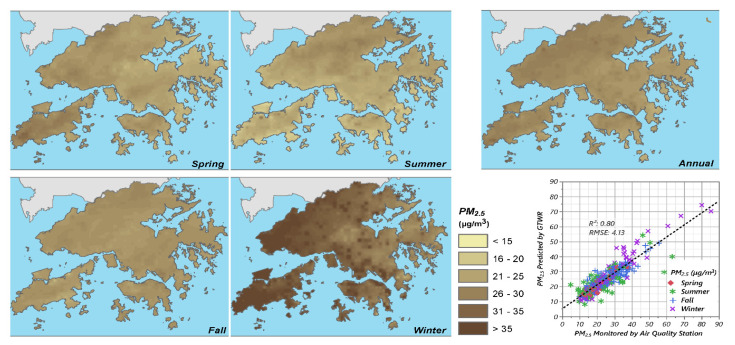
The four resulting maps of seasonal average and the map of the annual average of PM_2.5_ concentration level. The concentration value of 35 μg/m^3^ is the annual limit in Air Quality Objectives (AQOs) set out by the Air Pollution Control Ordinance (Cap. 311) of Hong Kong. The inset picture at the bottom right corner shows the actual predicted plot of the resulting models of PM_2.5_ concentration level.

The section reports the results from the first stage city-scale LUR. The LUR results include the resultant GTWR models and the seasonal maps of PM_2.5_. The structures of the four seasonal GTWR models are shown in Table 2, respectively. The estimation maps and corresponding regression plots are shown in Figure 2. The predictor variables selected by stepwise regression mainly reflect features that affect the PM_2.5_ level from the following three aspects: spatial location, built environment density, meteorological condition. For GTWR models, the coefficients were geographically and temporally varied (please refer to Table S4 of the supplementary material). AICc was calculated, which is the corrected version of the Akaike information criterion (AIC) [75]. Leave-one-out cross-validation (CV) which is commonly used for GWR-based model validation [76], was also performed to validate the model performance.

### 3.2. Neighborhood-Scale LUR Modeling Results

**Figure 3:**
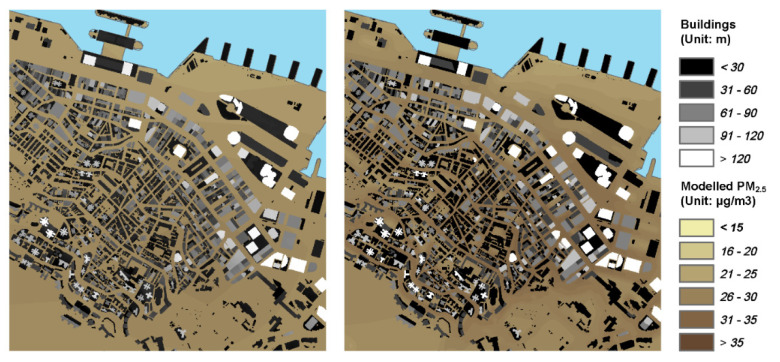
The zoom-in city-scale resulting PM_2.5_ map of the downtown area (left) with estimated fine-scale variability in PM_2.5_ ($\Delta PM_{2.5}$) overlayed (right).

The neighborhood-scale regression modeling was conducted by following the steps as stated in Section 2.4.2. The model structure and predictor coefficients are shown in Table 3. The model had a performance of $\bar{R^{2}}$ = 0.508, with all predictor variables having VIF < 4. The numerical range and summary statistics of input predictor variables of the neighborhood-scale LUR model are shown in Table S5 of the Supplementary Material. Figure 3 shows the zoom-in city-scale resulting PM_2.5_ map of the downtown area with estimated fine-scale variability in PM_2.5_ ($\Delta PM_{2.5}$) overlayed. More details of road space PM_2.5_ spatial variability are reflected.

## 4. Discussion

### 4.1. At the City-Scale

Built environment density determines the pollution emission as a denser area usually has a large population, and also affects the pollutant dispersal as the buildings alter the wind flow in the city. Indeed, a few studies indicate that a higher population density could mediate the balance between the usage of private and public transport [77], foster the implementation of cleaner technology [78], thereby reducing pollution emission. However, in most cases, evidence still shows that high population density is a main factor driving the accumulation of air pollution [79,80], which seems to be more in line with common perceptions. Such perception also appeared in the results of the present study. A statistically significant positive correlation between PM_2.5_ and residential land use area as well as the number of bus stops was observed in the resultant GTWR models. Spatial location, as another influential aspect, also showed in most models. Longitude was negatively correlated with PM_2_, while the correlation was opposite for Latitude. Such a pattern indicates a higher level of PM_2.5_ at northwest territories of Hong Kong, which represents the well-known air quality impact at the regional level [40]. The seasonal spatial maps also clearly showed the seasonal variability (Figure 2) [81]. The *FAI* calculated within a buffer radius of 250 m was identified as a major influential factor during summertime, which indicates the dominant effect of local building density and geometrical factors on the pollution dispersal in summer. Similar findings are also shown in other CFD simulation-based studies on the relationship between urban form and air pollution [82]. The critical buffer radius of 250 m for *FAI* is consistent with the findings from a previous study based on the long-term mobile ground-level air quality monitoring using the public transport vehicle platform [19]. The meteorological condition was also influential. Upper-air sounding indices related to atmospheric stability showed up in all seasonal models and shared the same relationship where higher atmospheric instability corresponded to a lower level of pollution concentration, vice versa. The reasonable explanation of the statistical model based on theoretical fundamental indicates the interpretability of the models. Compared with the annual model performance ($\bar{R^{2}}$ **=** 0.72) in our previous study on calibrating the AOD-PM_2.5_ relationship [68], the city-scale LUR model achieved better overall prediction performance ($\bar{R^{2}}$ **=** 0.80). Such a result further supports the conclusions of previous studies that the MODIS collection 6 MAIAC algorithm is more suitable than the 3 km MODIS AOD product for intraurban air quality studies [83]. Comparison between the present study results and the previous study results using 3 km MODIS also showed that the long-term PM_2.5_ concentration in Hong Kong during the period of 2018 to 2019 dropped by 46% compared to the years of 2003-2015 (with a reduction in annual average PM_2.5_ over the spatial extent of the entire city from 45.8 μg/m^3^ to 24.5 μg/m^3^). This reduction indicates the effectiveness and necessity of regional cooperative actions in air pollution control [84].

### 4.2. At the Neighborhood-Scale

In our previous study on calibrating the AOD-PM_2.5_ relationship [68], we mixed and put all predictor variables at various spatial scales into one single model. Although the previous models achieved moderate prediction performance, the models may still ignore the differences in the influence of predictors at various spatial scales on the dependent variables. The PM_2.5_ variations we captured during the in-situ sampling data are mainly determined by the environmental condition of the road segment. Therefore, the neighborhood-scale PM_2.5_ variation is supposed to be largely geolocation-independent, and thus should be investigated separately. In the present study, point-based building morphological indices were included in the model. It was found that point-based building morphological indices and road network centrality and accessibility metrics as predictors explained more than 50% of the variability in the in-situ PM_2.5_ sampling data. As indicated by the results shown in Table 3, road segments with higher connectivity and accessibility were found to be correlated to a higher level of PM_2.5_ concentration, as they usually hold more commercial activities and generally serve a larger traffic volume. The model also indicated that locations with higher openness generally correspond to lower levels of PM_2.5_ concentrations.

### 4.3. Limitations and Future Work

There is a limitation on AOD data filtering which should be noticed. As mentioned in Section 2.2.1, the AOD data used in this study is the MAIAC AOD. It has a finer resolution compared with the 3-km MODIS dataset but has more unusable pixels after applying the AOD quality filter. Usually, the QA filter “Best quality”, which combines the two filters: QA.CloudMask = “Clear” and QA.AdjacencyMask = “Clear”, should be used for most cases [31]. In this study, pixels with the filter of QA.CloudMask = “Possibly cloudy” were used to avoid the possible retrieval issue over certain urban areas [32]. In addition, pixels with the filter of QA for AOD = “AOD within ±2 km from the coastline” were retained to extend the spatial coverage of prediction. The above filtering scheme possibly introduced unreliable AOD data to the models. In that case, future work should focus on evaluating the uncertainties of the model. Moreover, future work might also be conducted in other testbed cities, which could acquire data outside the present study area and externally validate the present modeling and mapping results. Currently, the GTWR models are developed separately for different seasons. In future work, the model could be developed for various weather types by classifying meteorological conditions of pollutant dispersal. Another limitation of the present study is that the traffic was considered by introducing road network topological measures as the proxy. Although such methods have been employed by existing transport studies, using the traffic volume data from a computational traffic simulation conducted by transport engineers could significantly reduce the uncertainties and increase the neighborhood-scale regression model performance. Moreover, the regression model is only valid within the numerical range of input predictor variables as no model extrapolation was performed. Therefore, the estimation of neighborhood-scale PM_2.5_ variability does not have full spatial coverage of the city.

## 5. Conclusions

Investigating the multiscale complexity of spatial variability in air pollution is challenging, especially in compactly built environments. In this study, we tackled such multiscale complexity in the spatial variability of air pollution affecting pedestrian exposure levels by conducting in-situ sampling and multiscale LUR modeling of PM_2.5_, in the testbed city Hong Kong. Using the land-use regression (LUR) modeling technique as the fundamental method, air quality data, satellite data, meteorological data, and spatial data from multiple sources were integrated. We used a different strategy from existing LUR and AOD-PM_2.5_ research, which was to conduct the modeling at city- and neighborhood scales independently and examine the predictor variables at the two scales separately. At the city scale, the model developed in the present study obtained better prediction performance in the exploration of the AOD-PM_2.5_ relationship when compared with previous studies. At neighborhood scale, the point-based building morphological indices and road network centrality and accessibility metrics were found to be fit-for-purpose indicators of PM_2.5_ spatial estimation. The resultant PM_2.5_ map was developed by combining the models from the two scales. The map offers an estimation of intraurban variability of air pollution which facilitates local public health research. More importantly, working with practitioners and stakeholders, it is possible to convert the building morphological factors to urban planning and design language which could be incorporated into existing practice guidelines. Therefore, the quantitative information on the relationship between spatial predictors (e.g., building morphological factors) and PM_2.5_ concentration level derived from the models are interpretable and transferable to evidence-based strategies for the improvement in the built environment. This will enable knowledge transfer and potentially could inform the strategic planning direction of “planning for a livable high-density city” which is defined in the governmental initiative of planning vision and strategy of “Hong Kong 2030 +” by the Planning Department of Hong Kong [85]. More importantly, the methods and workflows proposed by the research have worldwide applicability, as all the necessary input data are not difficult to acquire in most regions.

## Figures and Tables

**Table 1:** Summary of the predictor variables and the corresponding data sources used in the multiscale LUR modeling in the present study.

Data Type	Predictor Variables	Unit	Abbr.	Raw Data Source	Spatial Scale
Weather data	Air temperature	*°C*	*TEMP*	Historical records are publicly accessible from local authorities of weather monitoring—Hong Kong Observatory (HKO)	Temporal-resolved variable with city-scale spatial variability
	Relative humidity	*%*	*RH*		
	Wind speed	*m/s*	*WSPD*		
	Rainfall	*mm*	*RF*		
	Mean sea level pressure	*hPa*	*MSLP*		
Atmospheric soundings	Sounding indices examined in this study are listed in Table S3 of the Supplementary Material			Wyoming Weather Web	Temporal variable
Land use *(Areal fraction of the land use type within certain circular buffer range ^1^)*	Residential land	*%*	*RES*	Derived from the open data from the Planning Department of Hong Kong (PlanD)	City scale
	Commercial land	*%*	*COM*		
	Industrial land	*%*	*IND*		
	Government land	*%*	*GOV*		
	Open space land	*%*	*OPN*		
	Greening cover ratio	*%*	*GCR*	Calculated based on above data	City scale
Geolocation of air quality monitoring stations and weather stations	Longitude	*degree*	*LONG*	The GeoInfo Map of Hong Kong	City scale
	Latitude	*degree*	*LAT*		
	Elevation above sea level	*m*	*ELEV*		
Population	Population density ^1^	*People/km^2^*	*POP*	WorldPop Global Project Population Data	City scale
Road network density *(For road: Line density of the land use type within certain circular buffer range ^1^; For bus station: the total number of stations within buffer)*	Trunk road/expressways	*km/km^2^*	*RD0*	Spatial data layers extracted from Open Street Map (OSM)	City scale
	Primary road	*km/km^2^*	*RD1*		
	Secondary road	*km/km^2^*	*RD2*		
	Tertiary road	*km/km^2^*	*RD3*		
	Ordinary road	*km/km^2^*	*RD4*		
	Bus stations	--	*BUS*		
Road segment attributes *(a set of metrics commonly used as a proxy of the road usage and traffic distribution)*	Normalized straightness	--	*STRAIGHT*	Calculation based on network centrality analysis	Neighborhood scale *(each road segment corresponds to a value)*
	Normalized betweenness	--	*BETWEEN*		
	Normalized closeness	--	*CLOSE*		
	Connectivity	--	*CONNECT*	Calculation based on Spatial Syntax	
	Control value	--	*CONTROL*		
	Mean depth	--	*MDEPTH*		
	Global integration	--	*GINTEG*		
	Local integration	--	*LINTEG*		
Building morphological data	Frontal area index ^1^	--	*FAI*	Calculated from the building dataset produced by Ren, et al. [42]	City scale
	Point-based FAI	--	*FAI_Point_*		Neighborhood scale
	Sky view factor	*[0–1]*	*SVF*		Neighborhood scale
	Surface roughness length	*m*	*ROUGHNESS*		City scale

^1^ For this spatial predictor variable, multiple values were calculated using a series of circular buffer radius: 50 m, 100 m, 200 m, 300 m, 400 m, 500 m, 750 m, 1000 m, 1500 m, 2000 m.

**Table 2:** Summary of the resultant GTWR model structures. The math operators before each predictor variable indicate its correlation with PM_2.5_. “+” indicates a positive correlation; “−” indicates a negative correlation. Model coefficients are shown in Table S4 of the supplementary material.

Season	Model Structure	$R^{2}$	$\bar{R^{2}}$	$CV\;R^{2}$	AICc
Spring	PM_2.5_ ~ AOD − LONG + KINX − PWAT	0.881	0.835	0.831	206.368
Summer	PM_2.5_ ~ AOD + FAI250 + LAT − KINX − PWAT	0.566	0.504	0.497	445.211
Fall	PM_2.5_ ~ AOD + RES500 + BUS400 + TEMP − WSPD + KINX − PWAT	0.772	0.694	0.673	1145.994
Winter	PM_2.5_ ~ AOD − LONG + ROUGHNESS50 + CINV + LCLP + LFCV + VTOT	0.898	0.853	0.846	579.051
Annual	A piecewise linear function is the combination of four seasonal models. Only one of the four models will be selected based on the time.	0.798	0.792	N.A.	N.A.

**Table 3:** Summary of the resultant neighborhood-scale regression model.

Predictor Variables	Coefficients	Significant Level	VIF
Intercept	−2.925 × 10^−1^	<0.0001	
Normalized betweenness	1.431 × 10^2^	<0.0001	1.009
Normalized closeness	3.074 × 10^5^	<0.0001	3.332
Control value	−1.675 × 10^−1^	<0.0001	2.050
Global integration	3.749	<0.0001	3.657
Sky view factor	−1.263 × 10^1^	<0.0001	1.959
Point-based *FAI*	2.826	<0.0001	1.716

## Data Availability

Not applicable.

## Supplementary Materials

The following are available online at https://www.mdpi.com/article/10.3390/ijerph19010321/s1, Figure S1: The maps and sampling routes in the ten study sites, Table S1: Detailed information of the 16 stations in the air quality monitoring network in Hong Kong, Table S2: The ten study sites selected for the PM_2.5_ sampling at neighborhood scale, Table S3: List of all atmospheric soundings indices examined in the stepwise regression modeling of this study, Table S4: Summary of coefficients of the four seasonal GTWR models, Table S5: The numerical range and summary statistics of input predictor variables of the neighborhood scale LUR model.
