# Supplementary material for: A Multiscale Land Use Regression Approach for Estimating Intraurban Spatial Variability of PM2.5 Concentration by Integrating Multisource Datasets
Source: Int J Environ Res Public Health. 2021 Dec 29;19(1):321. doi: 10.3390/ijerph19010321 (PMC8751171; doi:10.3390/ijerph19010321)
Supplement: Supplementary file 1 [file ijerph-19-00321-s001.zip › ijerph-1492589-supplementary.pdf]

Supplementary Materials of the Article -

A Multiscale Land Use Regression Approach for Estimating Intraurban Spatial Variability of $PM_{2.5}$ Concentration by Integrating Multisource Datasets

Yuan Shi [1,*], Alexis Kai-Hon Lau [2,3,4], Edward Ng [1,5,6], Hung Chak Ho [7], and Muhammad Bilal [8]

[1] Institute of Future Cities (IOFC), The Chinese University of Hong Kong, Hong Kong, China
[2] Division of Environment and Sustainability, Hong Kong University of Science and Technology, Clear Water Bay, Kowloon, Hong Kong, China; alau@ust.hk (A.K.-H.L.)
[3] Department of Civil and Environmental Engineering, The Hong Kong University of Science and Technology, Clear Water Bay, Kowloon, Hong Kong, China;
[4] Institute for the Environment, The Hong Kong University of Science & Technology, Clear Water Bay, Kowloon, Hong Kong, China
[5] School of Architecture, The Chinese University of Hong Kong, Hong Kong, China; edwardng@cuhk.edu.hk (E.N.)
[6] Institute of Environment, Energy and Sustainability (IEES), The Chinese University of Hong Kong, Hong Kong, China
[7] Department of Urban Planning and Design, The University of Hong Kong, Hong Kong, China; hcho21@hku.hk (H.C.H)
[8] Lab of Environmental Remote Sensing (LERS), School of Marine Sciences, Nanjing University of Information Science and Technology, Nanjing 210044, China; muhammad.bilal@connect.polyu.hk (M.B.)
* Correspondence: shiyuan@link.cuhk.edu.hk

This document contains supplementary material related to the research article titled "A multiscale land use regression approach for estimating intraurban spatial variability of $PM_{2.5}$ concentration by integrating multi-source datasets".

Table S1. Detailed information of the 16 stations in the air quality monitoring network in Hong Kong.

Region of the Station	*Latitude (degree)*	*Longitude (degree)*	*Station Types*	*Zoning in City Plan*	*Sampling Height Above Ground (m)*	*Building Height Average (m)*	*Building Coverage Ratio (%)*	*Sky View Factor*	*Green Space Coverage Ratio (%)*	*Road Area Ratio (%)*	*Road Network Density (km/km²)*	*Residential Land Use (%)*	*Commercial Land Use (%)*	*Governmental and Institutional Land Use (%)*	*Industrial Land Use (%)*	*Urban Open Space Percentage (%)*
CAUSEWAY BAY	22.2801	114.1851	Roadside	Urban Roadside	3	46.9	30.6	0.26	24.0	25.6	17.2	18.4	12.9	11.9	0.0	20.5
CENTRAL	22.2818	114.1581	Roadside	Urban Roadside	4.5	61.0	36.2	0.19	10.3	25.2	11.6	3.8	29.9	15.8	0.1	7.8
CENTRAL/ WESTERN	22.2849	114.1444	General	Urban	16	32.8	38.3	0.86	12.3	25.5	9.7	39.1	4.7	12.7	0.1	13.1
EASTERN	22.2829	114.2194	General	Urban	15	39.2	26.2	0.71	19.6	19.9	13.3	37.4	3.6	10.0	0.4	20.8
KWAI CHUNG	22.3571	114.1296	General	Urban	13	35.5	24.1	0.80	25.0	20.6	10.1	21.8	2.2	7.6	7.2	27.1
KWUN TONG	22.3096	114.2312	General	Urban	14.7	36.7	26.6	0.88	18.6	21.0	13.7	33.6	1.6	14.1	5.1	19.1
MONG KOK	22.3226	114.1683	Roadside	Urban Roadside	3	33.3	40.1	0.22	4.3	36.1	12.3	28.3	8.6	11.1	2.4	4.7
NORTH	22.4967	114.1282	General	New Town	23	27.7	12.2	0.87	14.7	21.4	9.5	25.5	0.0	17.9	0.3	13.3
SHA TIN	22.3763	114.1845	General	New Town	25	20.2	12.6	0.08	31.3	18.5	9.2	17.3	0.2	10.8	3.6	31.3
SHAM SHUI PO	22.3302	114.1591	General	Urban	17	28.6	32.2	0.22	8.0	28.1	11.0	40.6	1.5	7.5	1.2	7.9
SOUTHERN	22.2475	114.1601	General	Urban	18	30.0	14.2	0.88	30.4	12.3	6.0	8.4	0.5	13.3	4.5	30.6
TAI PO	22.4510	114.1646	General	New Town	28	24.8	25.6	0.95	18.0	17.6	6.3	38.3	0.9	11.4	0.0	17.2
TAP MUN	22.4713	114.3607	General	Rural	11	5.2	0.9	0.45	61.8	0.0	0.0	4.0	0.0	0.4	0.0	65.6
TSEUNG KWAN O	22.3176	114.2596	General	Urban	16	34.9	14.2	0.87	21.8	20.2	11.9	21.9	0.0	18.1	0.3	19.6
TSUEN WAN	22.3717	114.1145	General	Urban	17	36.4	32.6	0.85	6.5	24.1	9.5	24.4	3.9	8.1	3.9	6.5
TUEN MUN	22.3911	113.9767	General	New Town	27	24.9	16.6	0.85	26.9	20.2	11.1	33.5	0.7	12.0	0.2	26.4
TUNG CHUNG	22.2889	113.9437	General	New Town	27.5	36.6	11.9	0.90	29.6	20.9	8.9	28.8	4.3	4.1	0.0	24.6
YUEN LONG	22.4452	114.0226	General	New Town	25	19.3	22.7	0.97	20.7	19.1	7.4	35.8	0.9	12.9	0.2	17.7

Table S2. The ten study sites that selected for the $PM_{2.5}$ sampling at neighborhood scale.

Selected site	*Site No.*	*Abbr. of site names*	*Location[1] and terrain features*	*Function*	*Building density and morphology*	*Road network layout[2]*	*Air quality monitoring[3]*	*Measurement date (YYYYMMDD)*
Central	1	CEN	HKI, Coastal, Slight slope	Commercial	Compact high-rise	Irregular road network, express and trunk roads included.	Roadside	20190523
Causeway Bay	2	CSB	HKI, Coastal, Flat terrain	Commercial	Compact high-rise	Irregular road network, express and trunk roads included.	Roadside	20180618<br>20180711
North Point	3	NP	HKI, Coastal, Flat terrain	Governmental, Residential	Compact high-rise	Irregular road network, express and trunk roads included.	n.a.	20180612
Tsim Sha Tsui	4	TST	KL, Coastal, Flat terrain	Commercial, Residential, Sightseeing	Compact high-rise	Irregular road network, mainly primary and ordinary roads.	n.a.	20180517<br>20180722
Yau Ma Tei	5	YMT	KL, Flat terrain	Largely residential, partially commercial	Compact high-rise	Orthogonal grid road network, mainly primary and ordinary roads.	n.a.	20180915
Mong Kok	6	MK	KL, Flat terrain	Commercial, Residential, Sightseeing	Compact high-rise	Orthogonal grid road network, mainly primary and ordinary roads.	Roadside	20180518<br>20180725
Sham Shui Po	7	SSP	KL, Flat terrain	Largely residential	Compact high-rise	Orthogonal grid road network, mainly primary and ordinary roads.	General	20190513
Shek Kip Mei	8	SKM	KL, Flat terrain	Residential	Open-mid rise	Sparse road network layout, mainly ordinary roads.	n.a.	20190512
Kwun Tong	9	KTG	KL, Flat terrain	Industrial	Compact high-rise	Orthogonal grid road network, mainly secondary and ordinary roads.	General	20180603
Sha Tin	10	SHA	NT, Flat terrain	Residential, Open space	Open-high rise, Open-mid rise	Relatively sparse road network layout, mainly ordinary roads.	General	20180929

Note[1]: HKI – Hong Kong Island; KL – Kowloon; NT – New Territories.

Note[2]: Description of the road classes is based on OpenStreetMap (OSM).

Note[3]: Two types of long-term air quality monitoring stations of HKEPD – general station and roadside station.

Table S3. List of all atmospheric soundings indices examined in the stepwise regression modelling of this study (based on http://weather.uwyo.edu/upperair/indices.html).

Atmospheric soundings index	*Unit*	*Abbr.*
Bulk Richardson Number	-	*BRCH*
Bulk Richardson Number using CAPV	-	*BRCV*
Convective Available Potential Energy	*J/kg*	*CAPE*
CAPE using virtual temperature	*J/kg*	*CAPV*
Convective Inhibition	*J/kg*	*CINS*
CINS using virtual temperature	*J/kg*	*CINV*
Cross totals index	-	*CTOT*
K index	-	*KINX*
Pressure of the Lifted Condensation Level	*hPa*	*LCLP*
Temperature of the Lifted Condensation Level	*K*	*LCLT*
Level of Free Convection	*hPa*	*LFCT*
LFCT computed by using the virtual temperature	*hPa*	*LFCV*
Lifted index	-	*LIFT*
LIFT computed using virtual temperature	-	*LIFV*
Mean mixed layer mixing ratio	*g/kg*	*MLMR*
Mean mixed layer potential temperature	*K*	*MLPT*
Total precipitable water	*mm*	*PWAT*
Showalter index	-	*SHOW*
SWEAT index	-	*SWET*
Total totals index	-	*TTOT*
Vertical totals index	-	*VTOT*

Table S4. Summary of coefficients of the four seasonal GTWR models. The model structures are shown in Table 2 of main text.

	Mean	*Min*	*Quantiles 10%*	*Quantiles 25%*	*Median*	*Quantiles 75%*	*Quantiles 90%*	*Max*
				Spring GTWR model				
Intercept	3.287E+03	2.340E+03	2.418E+03	3.136E+03	3.348E+03	3.613E+03	3.704E+03	3.716E+03
AOD	2.603E+01	1.776E+01	1.826E+01	2.298E+01	2.622E+01	3.035E+01	3.062E+01	3.067E+01
LONG	-2.887E+01	-3.318E+01	-3.307E+01	-3.231E+01	-2.994E+01	-2.700E+01	-2.066E+01	-1.997E+01
KINX	7.302E-01	-1.244E+00	-1.241E+00	-1.230E+00	1.564E+00	2.541E+00	2.585E+00	2.634E+00
PWAT	-5.916E-01	-9.225E-01	-9.101E-01	-8.974E-01	-6.048E-01	-3.053E-01	-2.378E-01	-2.320E-01
				Summer GTWR model				
Intercept	-1.440E+03	-1.550E+03	-1.549E+03	-1.549E+03	-1.548E+03	-1.280E+03	-1.279E+03	-1.278E+03
AOD	4.478E+01	4.473E+01	4.473E+01	4.473E+01	4.474E+01	4.483E+01	4.484E+01	4.487E+01
FAI250	1.295E+01	1.072E+01	1.075E+01	1.079E+01	1.439E+01	1.440E+01	1.440E+01	1.440E+01
LAT	6.606E+01	5.878E+01	5.881E+01	5.884E+01	7.090E+01	7.096E+01	7.099E+01	7.100E+01
KINX	-3.978E-01	-4.110E-01	-4.108E-01	-4.108E-01	-4.106E-01	-3.782E-01	-3.775E-01	-3.771E-01
PWAT	-5.201E-01	-5.382E-01	-5.382E-01	-5.381E-01	-5.377E-01	-4.932E-01	-4.926E-01	-4.922E-01
				Fall GTWR model				
Intercept	1.028E+01	-2.027E+01	-1.627E+01	-2.290E+00	8.182E+00	2.191E+01	4.328E+01	4.882E+01
AOD	1.429E+01	-1.528E+01	-2.984E+00	-3.052E-02	1.357E+01	3.359E+01	3.753E+01	4.877E+01
RES500	1.403E-05	-5.305E-06	1.112E-06	1.290E-05	1.554E-05	1.768E-05	2.069E-05	2.716E-05
BUS400	1.041E-01	5.386E-02	6.076E-02	9.442E-02	1.013E-01	1.271E-01	1.527E-01	1.588E-01
TEMP	5.942E-01	-1.467E+00	-1.219E+00	2.771E-01	8.852E-01	1.360E+00	1.563E+00	1.826E+00
WSPD	-2.034E-01	-5.954E-01	-4.188E-01	-3.255E-01	-2.517E-01	-3.273E-02	7.078E-02	2.256E-01
KINX	8.586E-02	-2.964E-01	-1.007E-01	-4.968E-02	1.078E-01	2.178E-01	2.906E-01	3.580E-01
PWAT	-2.577E-01	-7.563E-01	-4.728E-01	-3.305E-01	-2.283E-01	-1.140E-01	-6.709E-02	-2.388E-02
				Winter GTWR model				
Intercept	2.817E+03	1.213E+03	1.657E+03	1.887E+03	2.241E+03	3.149E+03	5.697E+03	5.884E+03
AOD	3.217E+01	7.441E+00	8.972E+00	2.220E+01	2.897E+01	4.000E+01	6.236E+01	6.673E+01
LONG	-2.771E+01	-5.429E+01	-5.267E+01	-3.322E+01	-2.200E+01	-1.929E+01	-1.504E+01	-1.460E+01
ROUGHNESS50	6.455E-01	4.503E-01	4.714E-01	5.313E-01	6.610E-01	6.831E-01	8.153E-01	9.583E-01
CINV	7.719E-02	-2.221E-01	-2.057E-01	-1.040E-01	1.356E-01	2.951E-01	3.133E-01	3.236E-01
LCLP	2.660E-01	-5.502E-02	-3.212E-02	1.707E-01	3.612E-01	3.695E-01	4.140E-01	4.831E-01
LFCV	7.391E-02	-8.213E-03	-4.424E-03	6.789E-03	8.073E-02	1.437E-01	1.575E-01	1.704E-01
VTOT	2.454E+00	-3.084E+00	-2.864E+00	-2.850E+00	4.024E+00	5.029E+00	6.327E+00	7.163E+00

Table S5. The numerical range and summary statistics of input predictor variables of the neighborhood scale LUR model.

	Min	*Quantiles 10*	*Quantiles 25*	*Mean*	*Quantiles 50*	*Quantiles 90*	*Max*	*Range*
Normalized betweenness	0.000E+00	0.000E+00	2.759E-05	1.320E-03	2.739E-04	2.946E-03	2.863E-02	2.863E-02
Normalized closeness	6.501E-05	6.651E-05	6.842E-05	7.555E-05	7.791E-05	8.418E-05	8.470E-05	1.969E-05
Control value	1.667E-01	8.174E-01	1.959E+00	5.587E+00	3.236E+00	1.334E+01	2.083E+01	2.067E+01
Global integration	1.045E+00	1.660E+00	1.876E+00	2.128E+00	2.039E+00	2.579E+00	3.137E+00	2.093E+00
Sky view factor	1.814E-03	1.140E-01	1.741E-01	2.943E-01	2.547E-01	5.527E-01	8.623E-01	8.605E-01
Point-based FAI	1.069E-01	4.158E-01	7.531E-01	8.990E-01	9.245E-01	1.261E+00	1.974E+00	1.867E+00

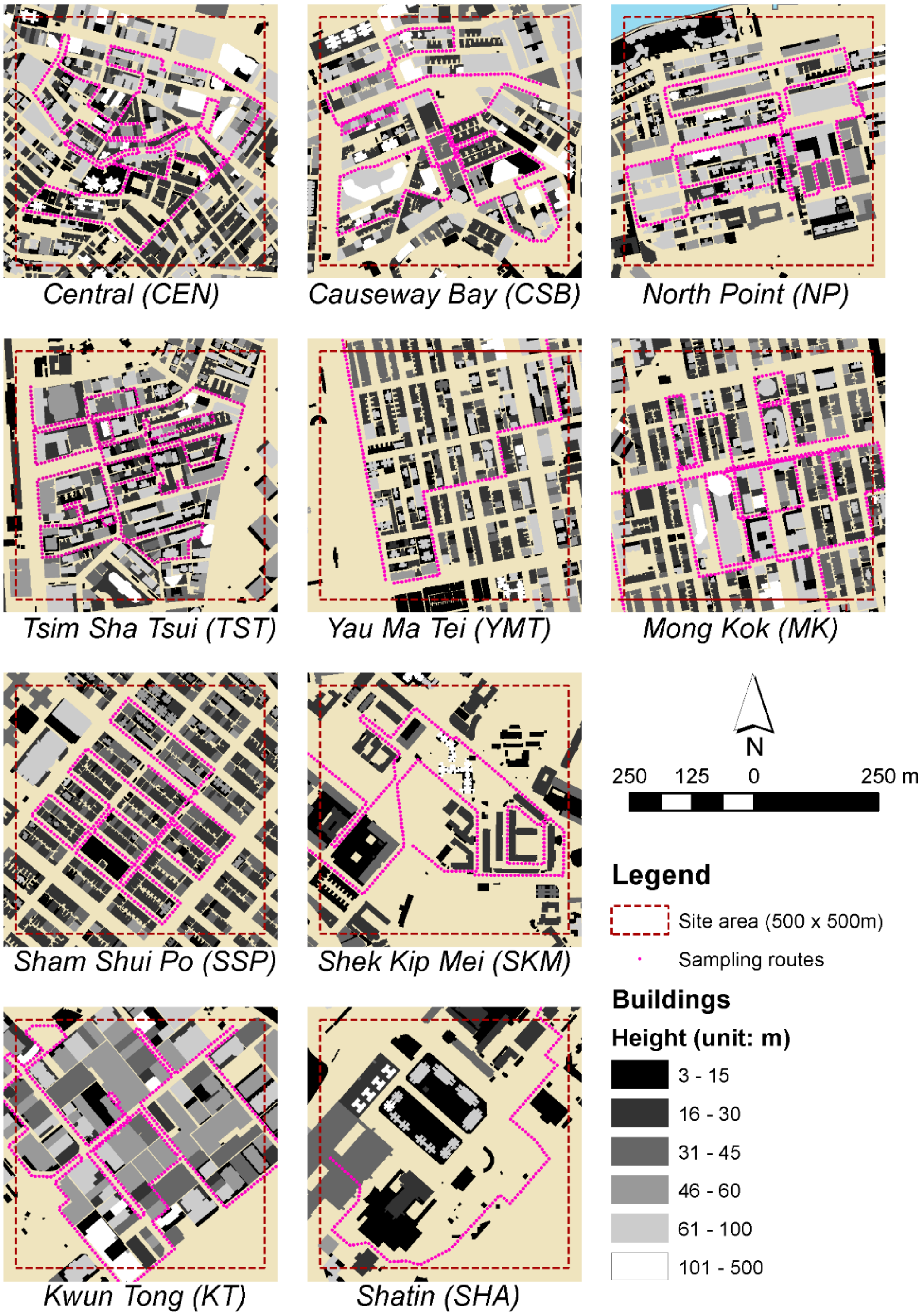

Figure S1. The maps and sampling routes in the ten study sites.
